# MicroRNA expression signature as a biomarker in the diagnosis of nodal T-cell lymphomas

**DOI:** 10.1186/s12935-024-03226-3

**Published:** 2024-01-30

**Authors:** Muhammad Sufyan Bin Masroni, Gracie Wee Ling Eng, Ah-Jung Jeon, Yuan Gao, He Cheng, Sai Mun Leong, Jit Kong Cheong, Susan Swee-Shan Hue, Soo Yong Tan

**Affiliations:** 1 Department of Pathology, National University Hospital, Singapore, 119077 Singapore; 2 Department of Pathology, Yong Loo Lin School of Medicine, National University of Singapore (NUS), Singapore, 117596 Singapore; 3 NUS Centre for Cancer Research, Singapore, 117599 Singapore; 4 Department of Biochemistry, Yong Loo Lin School of Medicine, National University of Singapore (NUS), Singapore, 117596 Singapore; 5 MiRXES Lab Pte Ltd, Singapore, 138667 Singapore; 6 Advanced Molecular Pathology Laboratory, Institute of Molecular and Cell Biology, Agency for Science, Technology and Research (A*STAR), Singapore, Singapore, 138673 Singapore

**Keywords:** miRNAs, T-cell lymphoma, Biomarkers, Nodal T-cell lymphoma, FFPE

## Abstract

**Background:**

The diagnosis of T-cell lymphomas is typically established through a multiparameter approach that combines clinical, morphologic, immunophenotypic, and genetic features, utilizing a variety of histopathologic and molecular techniques. However, accurate diagnosis of such lymphomas and distinguishing them from reactive lymph nodes remains challenging due to their low prevalence and heterogeneous features, hence limiting the confidence of pathologists. We investigated the use of microRNA (miRNA) expression signatures as an adjunctive tool in the diagnosis and classification of T-cell lymphomas that involve lymph nodes. This study seeks to distinguish reactive lymph nodes (RLN) from two types of frequently occurring nodal T-cell lymphomas: nodal T-follicular helper (TFH) cell lymphomas (nTFHL) and peripheral T-cell lymphomas, not otherwise specified (nPTCL).

**Methods:**

From the formalin-fixed paraffin-embedded (FFPE) samples from a cohort of 88 subjects, 246 miRNAs were quantified and analyzed by differential expression. Two-class logistic regression and random forest plot models were built to distinguish RLN from the nodal T-cell lymphomas. Gene set enrichment analysis was performed on the target genes of the miRNA to identify pathways and transcription factors that may be regulated by the differentially expressed miRNAs in each subtype.

**Results:**

Using logistic regression analysis, we identified miRNA signatures that can distinguish RLN from nodal T-cell lymphomas (AUC of 0.92 ± 0.05), from nTFHL (AUC of 0.94 ± 0.05) and from nPTCL (AUC of 0.94 ± 0.08). Random forest plot modelling was also capable of distinguishing between RLN and nodal T-cell lymphomas, but performed worse than logistic regression. However, the miRNA signatures are not able to discriminate between nTFHL and nPTCL, owing to large similarity in miRNA expression patterns. Bioinformatic analysis of the gene targets of unique miRNA expression revealed the enrichment of both known and potentially understudied signalling pathways and genes in such lymphomas.

**Conclusion:**

This study suggests that miRNA biomarkers may serve as a promising, cost-effective tool to aid the diagnosis of nodal T-cell lymphomas, which can be challenging. Bioinformatic analysis of differentially expressed miRNAs revealed both relevant or understudied signalling pathways that may contribute to the progression and development of each T-cell lymphoma entity. This may help us gain further insight into the biology of T-cell lymphomagenesis.

**Supplementary Information:**

The online version contains supplementary material available at 10.1186/s12935-024-03226-3.

## Introduction

T-cell lymphomas are a distinct subgroup of non-Hodgkin lymphomas (NHLs) that originate from mature T cells, accounting for approximately 10–15% of all NHL cases [1]. The most common clinical presentation of T-cell lymphomas is lymphadenopathy, or the abnormal enlargement of lymph nodes. The diagnosis of T-cell lymphoma involving the lymph nodes is typically established through an integration of clinical, morphologic, immunophenotypic, and genetic data, utilizing techniques such as light microscopy, immunohistochemistry (IHC), flow cytometry (FC), and T-cell receptor (TCR) gene rearrangement studies [2]. However, despite the utilization of a multiparameter approach, recognizing specific T-cell lymphoma entities and distinguishing them from reactive lymph node conditions remains a challenging task. This is attributed to the low prevalence and heterogeneous immuno-morphological features of T-cell lymphomas, which could diminish the confidence of even the most experienced pathologists in making the correct diagnosis [2].

The most prevalent subtypes of T-cell lymphomas that involve lymph nodes are nodal T-follicular helper (TFH) cell lymphomas (nTFHL) and peripheral T-cell lymphomas, not otherwise specified (nPTCL-NOS, but abbreviated as nPTCL for this paper). Together, these subtypes account for over 70% of all T-cell lymphomas that commonly involve the lymph nodes. T-follicular helper cell lymphomas is a group of T-cell neoplasms of postulated TFH cell origin, as reflected by the expression of the TFH immunophenotype and gene expression signature [3, 4]. PTCL-NOS, on the other hand, are defined by their T-cell lineage but lack other more distinctive features of specific T-cell lymphoma entities [5, 6]. These subtypes encompass a wide spectrum of cellular composition, cytologic, and immunophenotypic features that overlap with each other as well as with reactive lymphoid processes, making molecular testing an indispensable part of the diagnostic work-up. Recent work has contributed to a better understanding of the immunophenotypic and genotypic landscape of T-cell lymphomas [7], which will be reflected in lymphoma classifications.

In this study, we aimed to investigate the diagnostic potential of microRNA (miRNA) expression signatures as an adjunctive ancillary test in the classification of nodal T-cell lymphomas. miRNAs are a family of small, evolutionarily conserved, non-coding RNA molecules [8, 9] that have been shown to be promising diagnostic biomarkers. Because individual miRNAs can potentially regulate thousands of target genes [10], they have been implicated in both normal physiological processes as a master regulator of post-transcriptional gene expression [11, 12] and in the development of diseases such as cancer [13]. miRNA expression profiling is emerging as a valuable tool for tumor classification due to the high stability of miRNAs in clinical tissue samples. To achieve this goal, we used a novel, high-throughput, quantitative real-time PCR (qPCR) platform [14, 15] to profile miRNA expression in formalin-fixed paraffin-embedded (FFPE) patient samples of nTFHL, nPTCL, and reactive lymph nodes (RLN). We aim to evaluate whether miRNA signatures derived from FFPE patient samples can be used to differentiate cases of reactive lymph node from T-cell lymphomas and effectively distinguish between cases of nTFHL and nPTCL. Additionally, we performed functional enrichment analysis to uncover possible mechanistic involvement of these predictive miRNAs in cancer- or lymphoma-related pathways to gain further insight into the biology of T-cell lymphomagenesis.

## Materials and methods

### Subject recruitment and sample collection

FFPE tissue samples from RLN and 2 histological subtypes of nodal T-cell lymphomas were included: nTFHL and nPTCL, based on diagnostic criteria as published in the World Health Organization (WHO) Classification [6] of Tumours of Haematopoietic and Lymphoid Tissues. Selected cases were reviewed by pathologists with experience in hematolymphoid pathology to verify the diagnosis. Institutional Review Board approval was obtained in accordance with the NHG’s Institutional Review Board (IRB) Guidelines.

A total of 88 subjects were included in this study, comprising 19 RLN, 49 nTFHL and 20 nPTCL samples. Tissue samples were obtained from the Department of Pathology, National University Hospital (NUH), Singapore. The percentage of tumor cells was estimated to be 50% or more for each sample. Samples were analyzed using miRNA expression profiling and the results were compared to the reference diagnosis.

### RNA isolation

10 μm FFPE sections were used to RNA isolation. Deparaffinization and de-crosslinking was done using the DWX, RFTL and RF buffers of the Catchgene FFPE miRNA kit (MR23050) in accordance with the manufacturer’s instructions. After incubation and phase separation, the aqueous phase was transferred to a fresh tube and subjected to RNA isolation using the Maxwell RSC miRNA Tissue kit (Promega, AS1460) and the Maxwell RSC system (Promega) in accordance with the manufacturer’s recommendation. A proprietary spike-in control RNAs (~ 20 nt, MiRXES) was added into the sample lysis buffer prior to RNA isolation. Total RNA quantity and quality was accessed using NanoDrop 2000 (Thermo-Fisher Scientific).

### miRNA reverse transcription, cDNA pre-amplification and qPCR

cDNA synthesis was performed in 4 multiplex pools. For each pool, 50 ng of the extracted RNAs was reverse transcribed to cDNAs (25 °C for 10 min, 30 °C for 10 min, 35 °C for 10 min, 40 °C for 10 min, followed by 95 °C for 5 min) by conformational restricted miRNA specific-RT primers and the ID3EAL cDNA Synthesis System (MiRXES) on a SimpliAmp thermocycler (Applied Biosystems). cDNAs were subsequently split into 8 multiplex pools and preamplified ( 25 °C for 10 s, 95 °C for 10 min, 40 °C for 5 min, followed by 8 cycles of 95 °C for 10 s and 60 °C for 30 s) by the ID3EAL miRNA Augmentation System (MiRXES) using SimpliAmp Thermocycler (Applied Biosystems). qPCR master mix was added to preamplified cDNAs from each sample and precisely aliquoted to the ID3EAL PanoramiR microRNA Knowledge Panel (MiRXES, FGS0003) via the Integra Assist Plus liquid handler, prior to quantitative PCR (qPCR)-based miRNA expression profiling using the QuantStudio 5-384-well qPCR system (Applied Biosystems).

Upon the completion of miRNA expression profiling, raw threshold cycle (Ct) values were determined using the QuantStudio Design and Analysis software with automatic baseline and threshold settings. Technical variations introduced during RNA isolation and the process of RT-qPCR were normalized using the spike-in control RNA (ID3EAL PanoramiR Spike-in RNA Template, MiRXES).

### Data processing and statistical analysis

MiRNAs that were found to be expressed in less than 10% of the samples were first removed from our data analysis. The Ct values of miRNAs from each sample were further normalized to the global miRNA expression level. For each pairwise comparison group to RLN samples, log-2 transformed fold changes of the normalized miRNA expression level were used together with Student’s t-test. *P*-values were calculated with false discovery rate (FDR) adjustment. Benjamini and Hochberg method was used for FDR adjustment.

### Model building

2-class logistic regression was performed with a nested cross-validation training method. The model was built with 10-fold inner loop and evaluated with 5-fold outer loop.

Random forest model was also built with a nested cross-validation training method. The model was built with a 3-fold inner loop and evaluated with a 5-fold outer loop. Confusion matrix was tabulated from the average number from the 5 validation rounds. Through permutation-based feature selection, key miRNA features were identified.

In the nested cross-validation of both logistic regression and random forest models, the 10-fold inner loop was performed to tune the parameters of the model. After building the parameter space, grid search with elastic net was used to test different parameter combination. AUC was used to evaluate the performance with each parameter combination. The parameter combination with the highest AUC was selected. This was followed by the 5-fold outer loop to evaluate the model with the selected parameter combination. The final model performance was summarised with average AUC scores with 95% confidence intervals.

In logistic regression model, the following parameters were optimized in grid search :

1. Percentage of selected features : 5,10,20%.
2. Inverse of regularization strength : 0.0001, 0.001, 0.01, 0.1, 1, 10, 100, 1000, 10,000.
3. L1 ratio : 0, 0.25, 0.5, 0.75, 1.

In random forest model, the following parameters were optimized in grid search :

1. The maximum depth of the tree: 2,3,4.
2. Bootstraping : true, false.
3. The number of features to consider when looking for the best split : auto, sqrt, log2, none.
4. The function to measure the quality of a split: gini, entropy.

### Gene set enrichment analysis for miRNA target genes

Associations between miRNAs and mRNAs that have strong experimental evidences were curated with miRTarBase release 9.0. Target genes or genes associated with differentially expressed miRNAs were first identified, followed by pathways associated with those genes. Briefly, enrichment testing of pathways and transcription factors was performed based on hypergeometric distribution and Kolmogorov–Smirnov statistics. The *p*-value of enriched pathway was calculated by randomly permutating miRNAs, with false discovery rate correction.

## Results

### MiRNA expression profiling

**Fig. 1:**
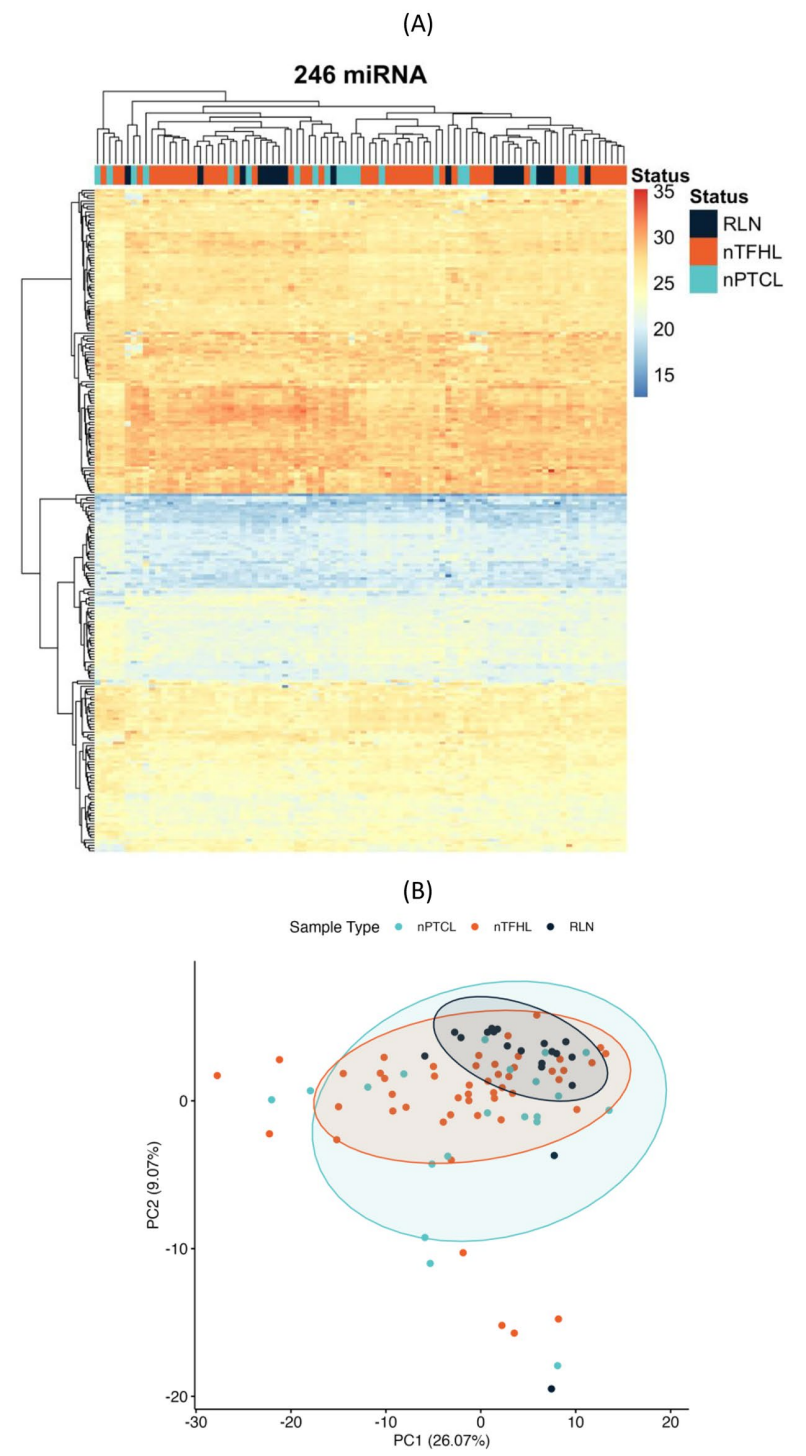
MiRNA expression profiling of T-cell lymphoma and reactive lymph node samples. (**A**) Heatmap showing hierarchical clustering of FFPE samples of nTFHL, nPTCL and RLN. (**B**) PCA plot capturing the variances in global miRNA expression for nTFHL, nPTCL and RLN samples

To assess miRNAs for their potential utility as diagnostic biomarkers for diagnosis and subtyping of nodal T-cell lymphomas using routine FFPE samples, we profiled a total of 88 samples using Mirxes’ curated panel of 376 high confidence mature miRNAs. During data processing, 130 miRNAs were removed due to the low detection rate. We performed hierarchical clustering (using euclidean distance and complete linkage) on the remaining 246 miRNAs and did not find distinct clusters for both subtypes of nodal T-cell lymphomas (Fig. 1A). Principal component analysis (PCA) captured 35% (PC1/2) of the total variance in miRNA expression in these subtypes (Fig. 1B).

### Differentially expressed miRNAs between reactive lymph nodes and T-cell lymphoma subtypes

**Fig. 2:**
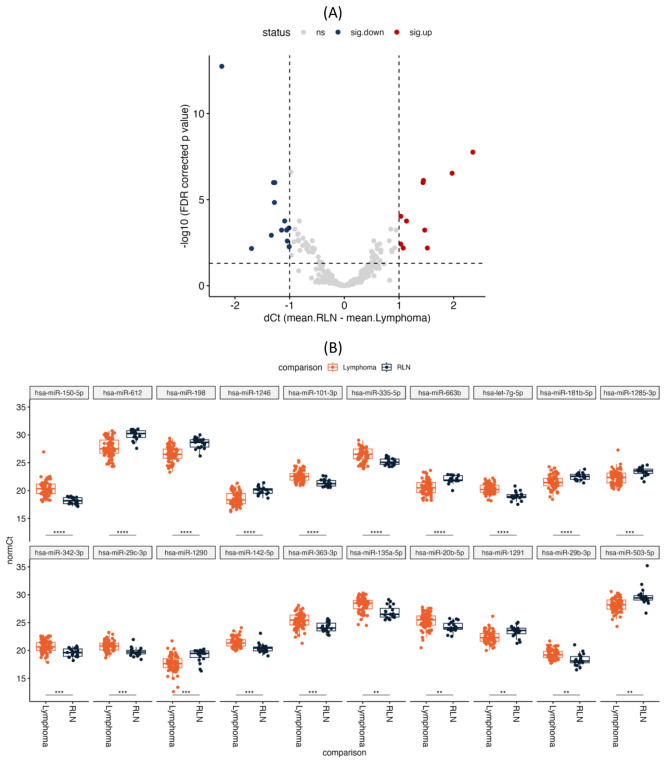
Differentially expressed miRNAs between T-cell lymphoma and reactive lymph node (RLN) samples. (**A**) Volcano plot showing the up- and downregulated miRNAs in all T-cell lymphomas as compared to RLN. (**B**) Box plots representing each differentially expressed miRNAs in T-cell lymphomas as compared to RLN. (** means *p*-value < 0.005, *** means *p*-value < 0.0005)

We performed a further analysis by comparing reactive lymph nodes (RL) with both lymphoma subtypes grouped together. We found 22 miRNAs that were differentially expressed with an FDR corrected *p*-value < 0.05 (Fig. 2A-B, Table S1). When comparing the individual subtypes of T-cell lymphoma against RL, nTFHL showed 18 differentially expressed miRNAs (9 downregulated and 9 upregulated miRNAs), while 25 miRNAs were deregulated in nPTCL (12 downregulated and 13 upregulated miRNAs) (Table S2-S3). All differentially expressed miRNAs identified in the grouped lymphoma analysis were also identified in either the nPTCL or nTFHL analyses or both. Notably, nPTCL has several unique miRNAs that were not identified in the grouped lymphoma analysis nor the nTFHL analysis.

### Logistic regression analysis of miRNA expression

**Fig. 3:**
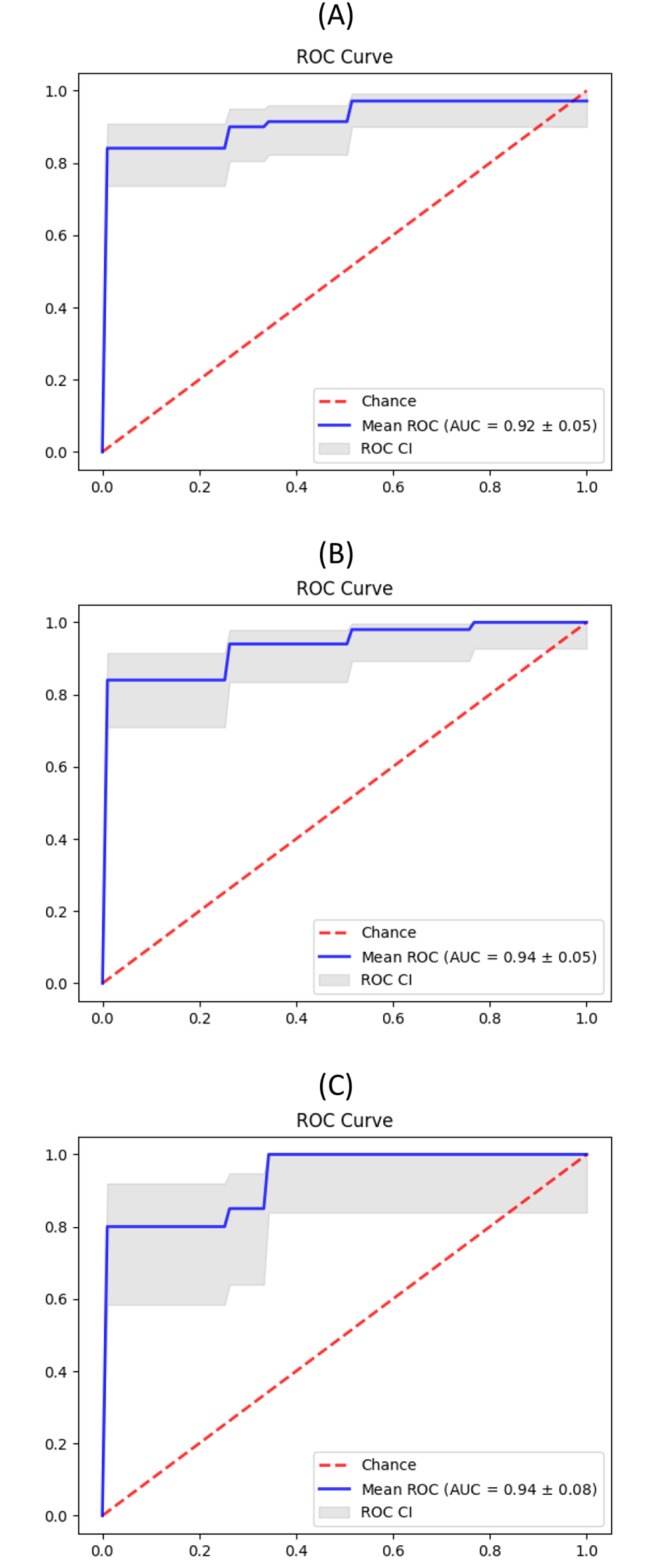
MiRNA expression as a classifier to distinguish reactive lymphoid nodes (RLN) from T-cell lymphomas. Two-class logistic regression was performed with a nested cross-validation training method, resulting in ROC curves in differentiating RLN from T-cell lymphomas (**A**), from nTFHL (**B**) and from nPTCL (**C**)

We then performed a new analysis using logistic regression (LR) with nested cross-validation as an alternative method to identify miRNAs that can differentiate the nodal T-lymphoma subtypes. We were able to identify subsets of miRNAs with a ROC-AUC of at least 0.92 (Table 1, Tables S4-S6, Fig. 3).

**Table 1:** Logistic regression analysis

	AUC	Best AUC	Number of miRNAs
RLN vs. T-cell lymphomas (nTFHL + nPTCL)	0.92 ± 0.05	0.935	49 (20%)
RLN vs. nTFHL	0.94 ± 0.05	0.95	13 (5%)
RLN vs. nPTCL	0.94 ± 0.08	0.95	25 (10%)

Table 1. Logistic regression analysis of miRNAs required to accurately differentiate between RLN and the comparator T-cell lymphoma subtype.

**Fig. 4:**
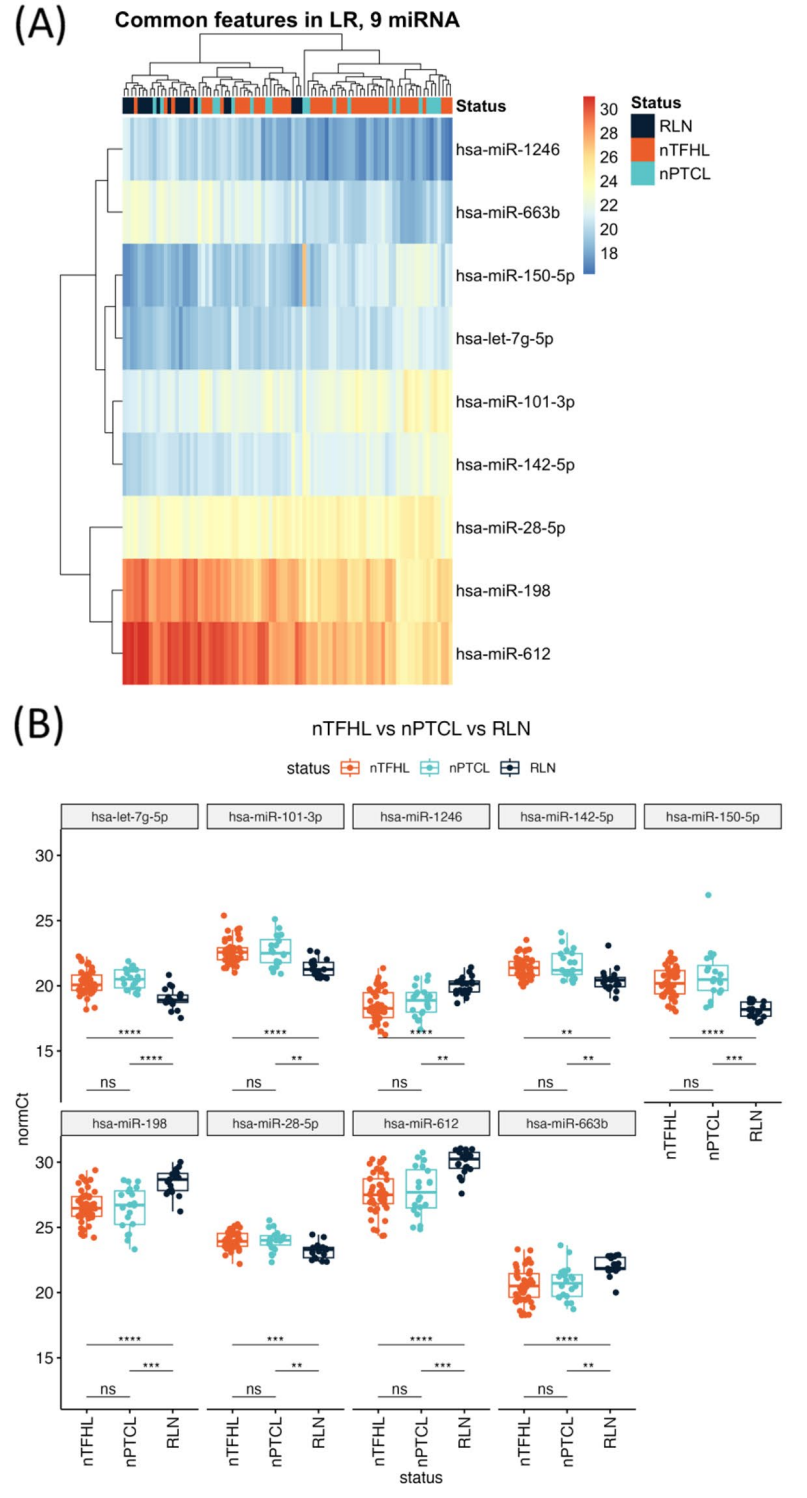
Common features in logistic regression analysis. (**A**) Heatmap showing hierarchical clustering of FFPE samples of nTFHL, nPTCL and RLN. (**B**) Box plots comparing the normalised Ct values of the miRNAs identified in logistic regression analysis. ns means not significant, ** means *p* < 0.01, *** means *p* < 0.001 and *** means *p* < 0.0001

Looking at the common features, a panel of 9 overlapping miRNAs were able to distinguish the different subtypes of T-cell lymphoma from RLN (Fig. 4A-B). Notably, a miRNA not identified in the earlier differential analysis, hsa-miR-28-5p, was identified in this panel.

### Random forest model

**Fig. 5:**
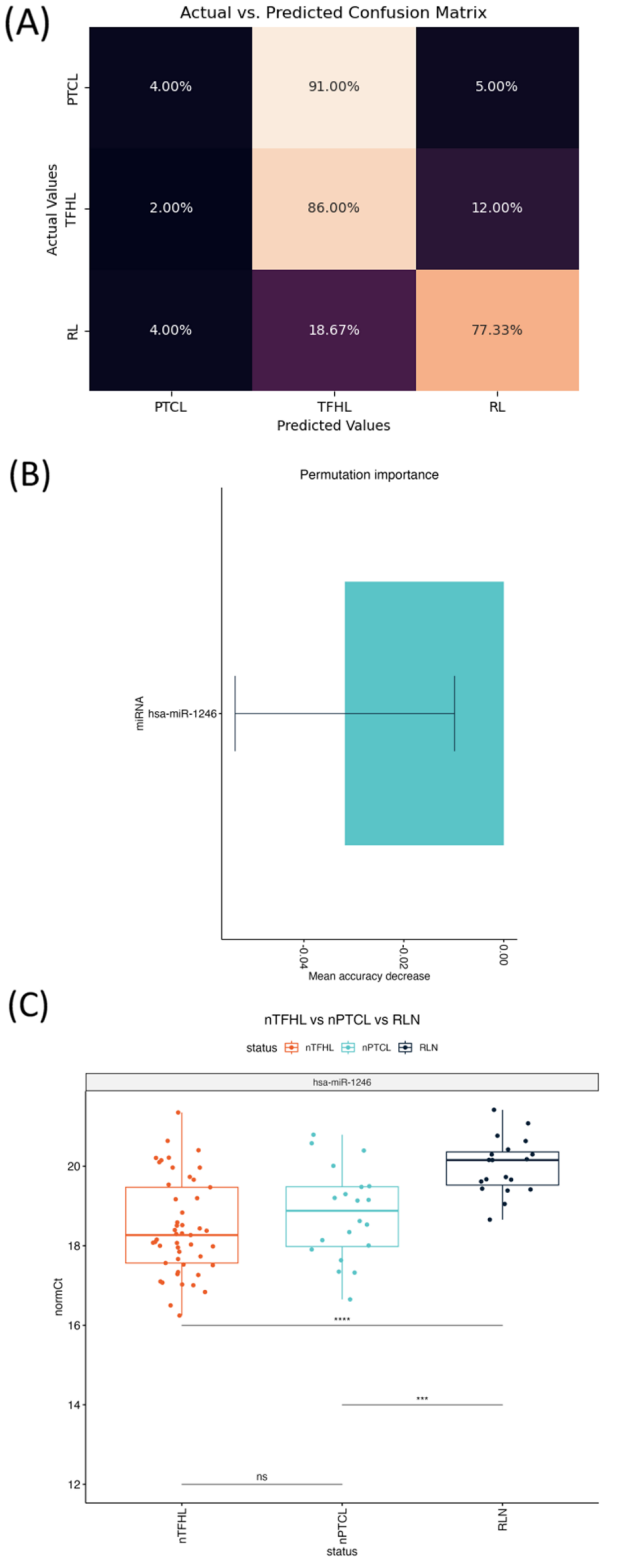
Random Forest model identified miRNAs that differentiate lymphomas from reactive lymph node. (**A**) Confusion matrix on the classification performance of the two nodal T-cell lymphoma subtypes based on the random forest model. (**B**) Permutation importance revealed the variable importance of miR-1246 as a differentiating miRNA. (**C**) Box plots comparing the normalised Ct values of miR-1246 in nTFHL, PTCL-NOS and RLN samples. ns means not significant, ** means *p* < 0.01, *** means *p* < 0.001 and *** means *p* < 0.0001

We have further utilized the random forest model, a powerful prediction algorithm that captures complex dependency patterns, to distinguish T-cell lymphomas from the reactive lymph nodes. Figure 5A presents the confusion matrix, illustrating the comparison between actual and predicted classes. Although the model successfully differentiated T-cell lymphoma samples from reactive lymph node samples, it encountered challenges in distinguishing between T-cell lymphoma subtypes. Particularly, differentiating nPTCL samples from nTFHL samples proved to be difficult. While the majority of the nTFHL samples (86.0%) were correctly predicted, only 4% of nPTCL samples were accurately classified, resulting in 91% of nPTCL samples being misclassified as nTFHL samples. This highlights the large similarity in miRNA expression patterns between the two lymphoma subtypes. The comparison of miRNAs between nPTCL and nTFHL showed no differentially expressed miRNAs, further supporting the observation that the T-cell lymphoma subtypes are highly similar in terms of their miRNA profiles (Supplementary Table S15).

To identify specific miRNAs responsible for these differentiating patterns, we performed permutation-based feature importance analysis and identified hsa-miR-1246 as a miRNA marker capable of distinguishing between reactive lymph nodes and lymphomas (Fig. 5B-C).

### MiRNA expression could infer biological differences between subtypes

**Fig. 6:**
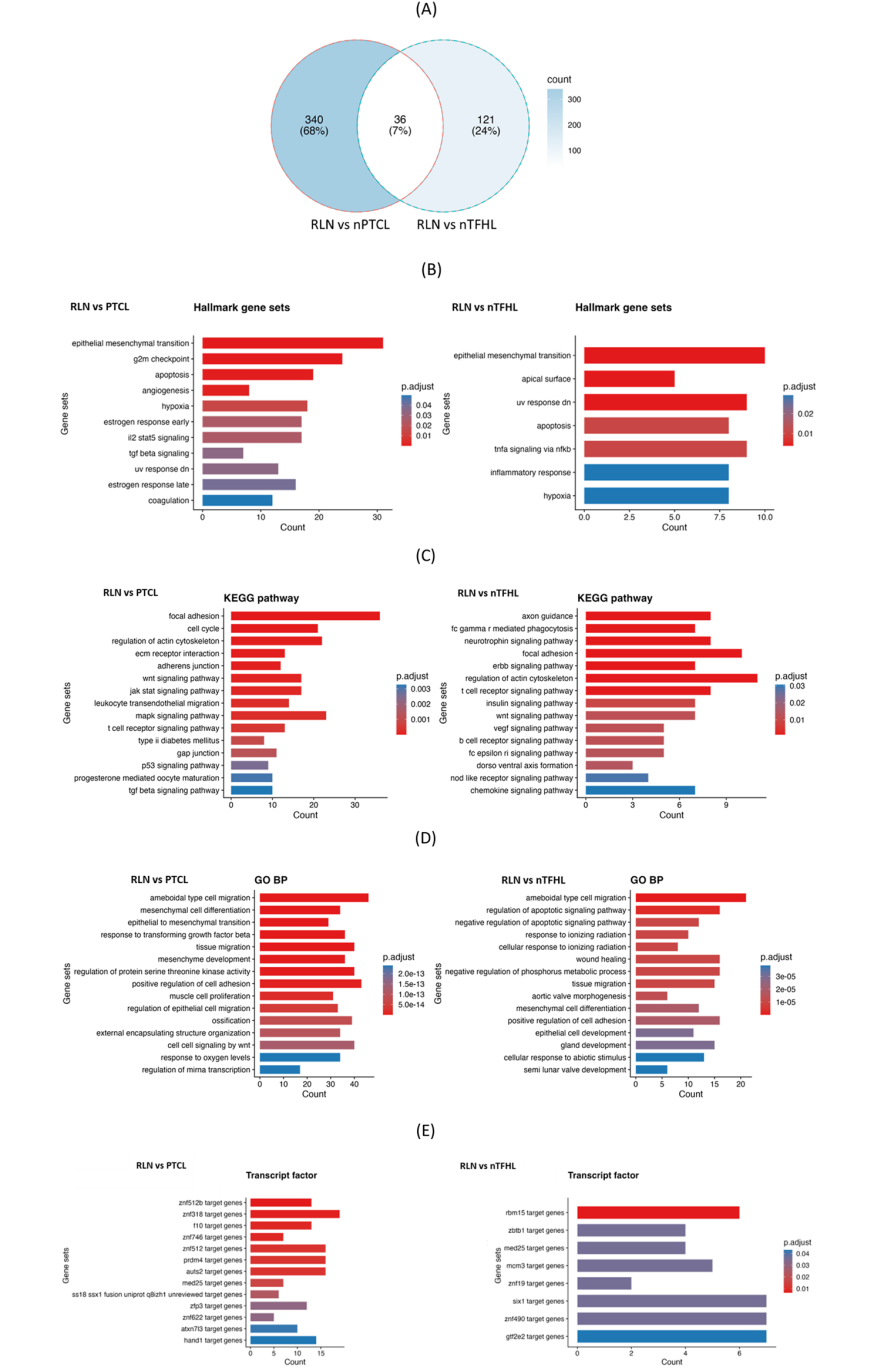
Gene set enrichment analysis of the miRNA target genes identifies biologically-relevant pathways and transcription factors in each subtype. (**A**) The target genes of unique differentially expressed miRNAs were first identified for each subtype (TFHL and PTCL-NOS). The number represents the target genes of the miRNAs uniquely found in each analysis. Bar charts showing enrichment for (**B**) Cancer Hallmarks gene sets, (**C**) KEGG pathway, (**D**) GO (Biological Process) pathways and (**E**) transcription factors. “Counts” refer to the number of miRNA target genes overlapping to the gene sets

We identified biological pathways and transcription factors that may be regulated by the differentially expressed miRNAs in each T-cell lymphoma subtype through the gene set enrichment analysis of the miRNA target genes. As nTFHL and nPTCL have been postulated to be part of a spectrum of cancers of T-cell origin, we identified the target genes of the dysregulated miRNA subsets unique to each subtype in order to pinpoint cellular pathways that may be driving each subtype (Fig. 6A). The gene set enrichment analysis of the miRNA target genes revealed the enrichment of multiple pathways known in literature to be dysregulated in nTFHL, such as the Tumor Necrosis Factor alpha (TNFα) signalling and inflammatory response pathways (Fig. 6B), Vascular endothelial growth factor (VEGF) signalling pathway (Fig. 6C) and negative regulation of the apoptotic pathway (Fig. 6D) [4].

## Discussion

### Current diagnostic challenges in T-cell lymphomas

The recognition of nodal involvement by T-cell lymphomas can pose a significant challenge for many practicing pathologists. The infiltration of neoplastic T cells can be difficult to detect and may resemble a reactive process due to mild cytological atypia and overlapping features. As such, an accurate diagnosis requires a comprehensive and meticulous morphological examination, immunophenotypic analysis, and, in many cases, an incorporation of clonality assessment. However, these diagnostic methods have several limitations and potential pitfalls, as outlined in Table 2.

**Table 2:** Current diagnostic challenges in T-cell lymphomas

Diagnostic parameter	Supportive features of T-cell lymphoma	Limitations and Pitfalls
Morphology	▪ Architectural effacement	▪ RLH can have architectural alterations (marked interfollicular expansion) that resemble T-cell lymphomas. ▪ Some T-cell lymphomas exhibit minimal changes to tissue architecture that can be similar to those seen in reactive hyperplasia.
	▪ Cytological atypia	▪ Cytologic atypia, particularly in nTFHL, may be minimal and display overlapping features with reactive T-cell proliferation. ▪ On the other hand, certain reactive lymph node conditions may present with increased numbers of large nucleolated (immunoblastic) cells that can be mistaken as neoplastic infiltrate.
Immunophenotype	▪ Aberrant T-cell immunophenotype, i.e., lost, or reduced expression of T-cell antigens. ▪ Aberrant pattern of expression of normal physiological markers e.g., CD30, TFH markers (PD1, ICOS, CXCL13) etc.	▪ Some T-cell lymphomas (particularly nTFHL) may retain full expression of T-cell antigens. ▪ Difficulty to interpret staining in small biopsy samples. ▪ Significant inter- and intra-observer variability. ▪ Quality of lab processing and IHC protocol will significantly affect results. ▪ A good understanding of protein biology and expression pattern is required for accurate interpretation.
Clonality	▪ Monoclonal TCRB or TCRG gene rearrangements in T-cell lymphomas.	▪ Monoclonal TCRB or TCRG rearrangements may be detected in reactive T-cell proliferations, for example in viral infections. ▪ Conversely, false negativity can occur in some T-cell lymphomas with significant reactive inflammatory background, and in small biopsy samples with low volume of neoplastic T-cells.

*RLH: Reactive lymphoid hyperplasia; nTFHL: nodal T follicular helper cell lymphoma; TFH: T follicular helper; IHC: Immunohistochemistry: TCRB: T cell receptor Beta; TCRG: T cell receptor Gamma

Due to the widespread demand for COVID-19 testing during the global pandemic, the availability of RT-qPCR machines has significantly increased. Therefore, we investigated the feasibility of using a carefully selected panel of miRNA-based PCR assays as a cost-effective and practical complement to traditional morphological diagnosis. Furthermore, miRNA biomarkers may also provide adjunctive molecular evidence to increase our confidence in distinguishing between reactive and neoplastic T-cell proliferation in pathological diagnoses.

In this cohort of nodal T-cell lymphomas, we measured 246 miRNAs and characterised miRNA biomarker profiles by differential expression, logarithm regression and random forest model. We found that the unique differentially expressed miRNAs implicates a variety of pathways which are both known players in the pathophysiology of nodal T-cell lymphomas and potentially novel, understudied pathways in this group of lymphoid malignancy.

### Biological relevance of miRNA biomarkers

We found a subset of common miRNAs across our analysis for differentially expressed miRNAs and logistic regression that have been implicated in the pathophysiology of lymphoma.

MiR-663b has been shown to be the top 3 upregulated miRNAs in cutaneous T-cell lymphoma [16], although its exact role in the pathophysiology of T-cell lymphomas remains unclear. We also observed its upregulation in our T-cell lymphoma samples, thus highlighting the relevance and reliability of our miRNA discovery approach.

Hsa-let-7 g has also been shown to be intricately involved in T cells. It is highly expressed in naïve T cells to maintain quiescence [17] and upon T cell activation, its reduction contributes to clonal expansion and an effector phenotype [18]. Hence, its downregulation in our T-cell lymphoma samples may play a role in their pathogenesis and aggressive phenotype.

MiR-101, which is upregulated in our samples, has been correlated with the Th2 phenotype and shown to regulate cell proliferation and apoptosis, albeit in other forms of lymphomas [19]. Hence, miR-101 may potentially shape the differentiation program and the proliferation rate of T-cell lymphomas.

MiR-198 overexpression in CD8 + T cells has been associated with dysfunctional immunity and increased apoptosis, although in the context of the renal cell carcinoma tumour microenvironment [20]. Its upregulation in our T-cell lymphoma samples may promote a similar pro-oncogenic mechanism.

MiR-142-5p is known to be a hematopoietic-specific miRNA that exclusively regulate T-cell response but not T-cell development, and its ablation reduces Graft versus Host Disease (GVHD) in murine models [21]. Its downregulation in our nodal T-cell lymphoma samples may provide to a mechanistic explanation for dysfunctional T-cell response.

MiR-150-5p is yet another critical miRNA with temporal regulatory role throughout the complex process of hematopeoisis and is frequently dysregulated in hematological malignancies. The downregulation of miR-150-5p in our samples appear to be consistent with the reported reduction of miR-150-5p expression in CD4 + T-cells when they differentiate into the Th1 and Th2 lineages during normal development [22].

MiR-28-5p is a major regulator of the germinal centre reaction and is hence more relevant in B-cell lymphomagenesis [23]. Its downregulation in our T-cell lymphoma samples suggests that miR-28-5p may also play a critical role in T-cell lineage determination.

To our knowledge, the remaining miRNA candidates that we have identified (such as miR-1246 and miR-612) have not been implicated or studied in the context of T-cell lymphoma. Future studies can be designed to functionally characterize their roles in promoting T-cell lymphoma development.

Overall, we demonstrate that a number of differentially expressed miRNAs discovered in this study have been implicated in various aspects of hematopoietic function and regulation and could play pivotal roles in nodal T-cell lymphoma.

Interestingly, the miRNA signatures identified in our study are not similar to other miRNA profiling studies performed on T-cell lymphomas [24–26]. There are notable contextual differences between these studies that could explain the differing signatures. Most significantly, our study specifically compared nodal lymphoma tissues to RLN, hence our miRNA signatures could be more contextualized to normal and transformed nodal tissues, whereas other studies generally compared T cell lymphomas to normal peripheral T cells. Laginestra et al. similarly studied nodal PTCL-NOS samples – however, the comparator was normal peripheral T cells, not RLN [26]. Another reason could be the differences in the selection criteria used for the study cohorts. For example, Lone et al. excluded cases with TFH phenotype from their miRNA profiling study on PTCL whereas nTFHL cases are well-represented in our study [24].

### Biological relevance of pathways implicated by miRNAs

Besides their potential utility as biomarkers, deregulated miRNAs in tumors may also help us to better understand the gene networks that underpin T-cell lymphomagenesis and progression. For instance, we performed pathway enrichment analysis on the target genes of unique differentially expressed miRNAs to predict key biological pathways in promoting T-cell lymphoma development and found that our results are consistent with reported literature.

TFHL have been well-characterised in terms of mutations and the pathways they affect, especially in in angioimmunoblastic T-cell lymphoma (AITL), a major subtype of nTFHL [27]. Frequent mutations that have been identified in AITL include, among others, RHOA, IDH2, TET2 and DNMT3A [4]. The TFHL gene expression signature has been reported to include, among others, TNFα signaling by NF_Κ_B and the production of T cell-induced cytokines (IL2, IL15 and IL17) [4]. In congruent to this, synonymous pathways – specifically TNFα signalling pathways and inflammatory response pathways (which covers pro-inflammatory cytokines) - are identified as hallmark gene sets enriched in nTFHL (Fig. 4b). These findings are supported by evidence in mice models, whereby loss of TET2 and RHOA G17V – both mutations with high co-occurrence frequency in AITL- contributed to increased TNFα levels to reinforce the T follicular helper (Tfh) signature [28]. The same study on co-occurring loss of TET2 and RHOA G17V also demonstrated an increase in VEGF-A signaling (also identified in Fig. 4c), and a decrease in the expression of the pro-apoptotic receptor gene, Fas, leading to the negative regulation of the apoptotic signaling pathway as captured by Gene Ontology (GO) enrichment analysis (Fig. 5D) [28]. Another pathway, MAPK signaling (Table S8), is linked to aberrant chromosomal gain associated with IDH2 R172 mutation [29] and in other cases, RHOA G17V mutation [28].

PTCL-NOS, on the other hand, is a group of entities that lacks distinctive features of specific T-cell lymphoma entities and are hence likely to feature a heterogeneous phenotypes featuring a wider range of pathways (Table S11-13).

Additionally, as miRNAs regulate essentially all gene-encoding RNA transcripts, including transcription factors, we could enhance the robustness of our pathway enrichment prediction by supplementing it with miRNA-transcription factor interactome. For instance, our analysis pointed to the enrichment of the target genes of ZBTB1, an important transcription factor in the development of conventional CD4/CD8 αβ + T cells [30], specifically in nTFHL samples. Interestingly, MED25 was commonly identified in both nTFHL and nPTCL, pointing to a possible unexplored role of this transcription factor in both lymphoma subtypes. MED25 is one of the many subunits of the co-regulator Mediator complex that engages RNA Pol II for general transcription. However, MED25 itself is required for the transcription initiation of the rate-limiting gene responsible for retinoic acid biosynthesis in macrophages [31]. As retinoic acid is also essential for peripheral induction of the helper T cell phenotype and and its proinflammatory response, we hypothesise that MED25 may be involved in T cell lymphomagenesis [32].

### Limitations of the study

Firstly, the results of our study should ideally be validated in a larger patient and normal cohort as our sample size was limited. Secondly, we used a curated panel of 376 miRNAs, which represents a portion of the entire miRNAome comprising 2,600 mature miRNAs [33]. Hence, we could be missing out on other biologically relevant miRNAs that may potentially distinguish between the nTFHL and nPTCL subtypes.

## Conclusions

Overall, our results demonstrate that miRNA expression profiling may serve as promising biomarkers and a practical tool to aid the diagnosis of nodal T-cell lymphoma, which can be challenging. Specifically, we explored three different methods to identify miRNA signatures that can help to differentiate nodal T-cell lymphomas (specifically nTFHL and nPTCL) from RLN. We found that miRNA signatures derived from logistic regression may potentially be used as a diagnostic adjunct in clinical classification of T-cell lymphoma cases from RLN. Using logistic regression, we identified miRNA signatures that can distinguish RLN from nodal T-cell lymphomas (AUC of 0.92 ± 0.05 using a 49-miRNAs signature), from nTFHL (AUC of 0.94 ± 0.05 using a 13-miRNAs signature) and from nPTCL (AUC of 0.94 ± 0.08 using a 25-miRNAs signature). However, because of the similarity of miRNA expression patterns between nTFHL and nPTCL, our miRNA signatures are not able to distinguish between the two subtypes. Bioinformatic analysis of the differentially expressed miRNAs also revealed relevant and potentially understudied signalling pathways that are unique to each T-cell lymphoma entity.

### Electronic supplementary material

Below is the link to the electronic supplementary material.

Supplementary Material 1

Supplementary Material 2

## Data Availability

The datasets used and/or analysed during the current study are available from the corresponding author on reasonable request.

## Abbreviations

AUC: Area under the curve
CML: Chronic myelogenous leukemia
EMT: Epithelial-mesenchymal transition
GO: Gene Ontology
LR: Logistic regression
MiRNA: microRNA
nPTCL: Nodal peripheral T-cell lymphomas, not otherwise specified
nTFHL: Nodal T-follicular helper (TFH) cell lymphomas
PCA: Principal Component Analysis
PTCL: Peripheral T-cell lymphomas
PTCL-NOS: Peripheral T-cell lymphomas, not otherwise specified
RLN: Reactive lymph node
ROC: Receiver Operating Characteristic Curve
TCR: T-cell receptor
Tfh: T follicular helper
TGFβ: Transforming growth factorβ
TNFα: Tumor necrosis factorα
VEGF: Vascular endothelial growth factor
