# Supplementary material for: MicroRNA expression signature as a biomarker in the diagnosis of nodal T-cell lymphomas
Source: Cancer Cell Int. 2024 Jan 30;24:48. doi: 10.1186/s12935-024-03226-3 (PMC10826179; doi:10.1186/s12935-024-03226-3)
Supplement: Supplementary file 2 — Supplementary Material 2 [file 12935_2024_3226_MOESM2_ESM.pdf]

Supplementary Methods

Model building

2-class logistic regression (with elastic net regularization) was performed with a nested cross-validation training method. The model was built with 10-fold inner loop and evaluated with 5-fold outer loop.

Random forest model was also built with a nested cross-validation training method. The model was built with a 3-fold inner loop and evaluated with a 5-fold outer loop. Confusion matrix was tabulated from the average number from the 5 validation rounds. Through permutation-based feature selection, key miRNA features were identified.

In the nested cross-validation of both logistic regression and random forest models, the 10-fold inner loop was performed to tune the parameters of the model. After building the parameter space, grid search with elastic net was used to test different parameter combinations. AUC was used to evaluate the performance with each parameter combination. The parameter combination with the highest AUC was selected. This was followed by the 5-fold outer loop to evaluate the model with the selected parameter combination. The final model performance was summarized with average AUC scores with 95% confidence intervals.

In logistic regression model with elastic net regularization, the following parameters were optimized in grid search :

1. Percentage of selected features : 5,10,20%. ANOVA test and F-value were used in the initial selection of features.

2. Inverse of regularization strength : 0.0001, 0.001, 0.01, 0.1, 1, 10, 100, 1000, 10000

3. Lasso regression was also considered. L1 ratio : 0, 0.25, 0.5, 0.75, 1

In random forest model, the following parameters were optimized in grid search :

1. The maximum depth of the tree: 2,3,4

2. Bootstraping : true, false

3. The number of features to consider when looking for the best split : auto, sqrt, log2, none

4. The function to measure the quality of a split: gini, entropy
